# A Novel and Efficient Method for Bacteria Genome Editing Employing both CRISPR/Cas9 and an Antibiotic Resistance Cassette

**DOI:** 10.3389/fmicb.2017.00812

**Published:** 2017-05-05

**Authors:** Hong Zhang, Qiu-Xiang Cheng, Ai-Min Liu, Guo-Ping Zhao, Jin Wang

**Affiliations:** ^1^Key Laboratory of Synthetic Biology, Institute of Plant Physiology and Ecology, Shanghai Institutes for Biological Sciences, Chinese Academy of SciencesShanghai, China; ^2^University of Chinese Academy of SciencesBeijing, China; ^3^Shanghai Tolo Biotechnology Company LimitedShanghai, China; ^4^Provincial Key Laboratories of Conservation and Utilization for Important Biological Resource and Biotic Environment and Ecological Safety, College of Life Sciences, Anhui Normal UniversityWuhu, China

**Keywords:** CRISPR/Cas9, protospacer adjacent motif, genome editing, antibiotic resistance cassette, sequence-independent

## Abstract

As Cas9-mediated cleavage requires both protospacer and protospacer adjacent motif (PAM) sequences, it is impossible to employ the CRISPR/Cas9 system to directly edit genomic sites without available PAM sequences nearby. Here, we optimized the CRISPR/Cas9 system and developed an innovative two-step strategy for efficient genome editing of any sites, which did not rely on the availability of PAM sequences. An antibiotic resistance cassette was employed as both a positive and a negative selection marker. By integrating the optimized two-plasmid CRISPR/Cas system and donor DNA, we achieved gene insertion and point mutation with high efficiency in *Escherichia coli*, and importantly, obtained clean mutants with no other unwanted mutations. Moreover, genome editing of essential genes was successfully achieved using this approach with a few modifications. Therefore, our newly developed method is PAM-independent and can be used to edit any genomic loci, and we hope this method can also be used for efficient genome editing in other organisms.

## Introduction

The practicability of targeted gene editing is important for understanding the biological functions of genes. To date, several homology-directed repair (HDR)-based genetic modification technologies have been developed in the model microorganism, *Escherichia coli*, and two-step strategies including a negative selection step make it possible to perform markerless genome editing (Datsenko and Wanner, 2000; Tischer et al., 2006; Peters et al., 2013; Wang et al., 2014). A large number of toxic genes have been verified to work well as negative selection markers in *E. coli*, including *sacB, ccdB* and *codAB* (Yu et al., 2008; Kostner et al., 2013; Wang et al., 2014). However, the efficiency is low during the second step of crossover to lose the toxic genes. Furthermore, spontaneous mutations may exist in the toxic genes during the counter-selection process, generating false positives. Therefore, a general and accessible method for the modification of genomic sequences of interest in bacteria would be of great value in multiple applications such as metabolic engineering.

CRISPR is an adaptive immunity system in prokaryotes that provides specific resistance against infection by bacteriophage (Bolotin et al., 2005; Deveau et al., 2008). The CRISPR system has been harnessed by scientists to create powerful RNA-guided genome editing tools, and this technology has been widely applied in various research fields (Cong et al., 2013; Hwang and Al, 2013; Jiang et al., 2013; Jinek et al., 2013). With the development of CRISPR/Cas9 technologies, Cas9 can be used to introduce double-stranded DNA (dsDNA) breaks at target DNA sequences and only cells for which the target sequences have been edited (i.e., through HDR with donor DNA) can survive, thus increasing the genome editing efficiency. Following this principle, Jiang et al. (2015) recently reported the use of the CRISPR/Cas9 system to perform multigene editing in *E. coli*. Notably, the protospacer adjacent motif (PAM) and the protospacer sequences must be removed from the edited target sequences; otherwise, the target protospacer sequences will be continuously recognized and cleaved by Cas9, which may lead to cell death. Importantly, in most cases (e.g., point mutations), the target site to be mutated does not locate within the PAM or the protospacer sequences. Although this problem can be solved by the introduction of mutations or deletions into the PAM and (or) protospacer sequences at the same time (Bao et al., 2015; Li et al., 2015; Mans et al., 2015; Pyne et al., 2015; Bassalo et al., 2016; Zhao et al., 2016), new mutations will be generated, which may influence subsequent analysis and deflect from the goal of seamless and precise genome editing.

**FIGURE 1:**
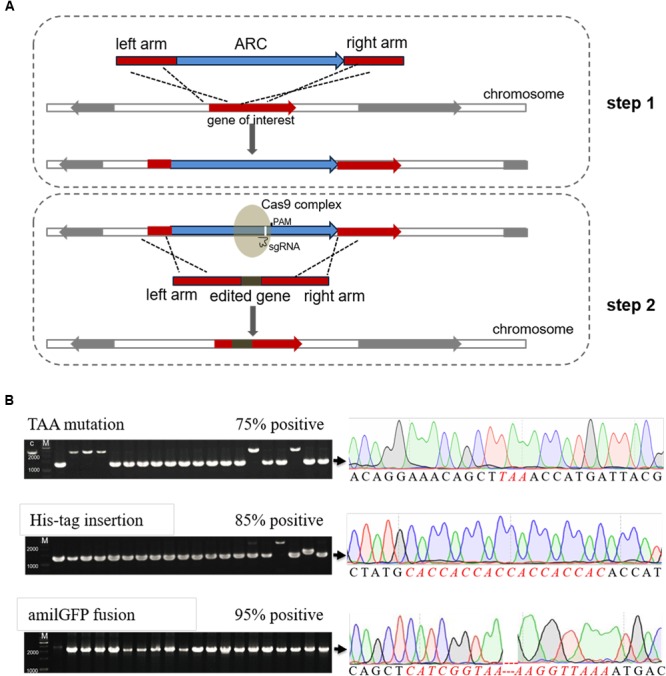
**Efficient genome editing by employing both an antibiotic resistance cassette (ARC) and the CRISPR/Cas9 system. (A)** Schematic chart illustrating the two-step strategy for editing non-essential genes (or any DNA sequences) in *E. coli*. Firstly, an ARC was inserted into the genome near to the target site through homology-directed repair (HDR). Secondly, the Cas9 complex targeting the ARC sequence cleaved the ARC, and meanwhile the ARC was replaced with the edited sequence through the second step of HDR. The target sequences could be mutated, deleted or inserted with foreign DNA sequences, and the λ-Red recombination system remarkably increased the HDR efficiency in both steps. **(B)** Verification of the edited mutants by both colony PCR (left) and Sanger DNA sequencing (right). Correct sizes are indicated at the end of arrows, and the positive rates are labeled. The PCR products that formed positive clones were then randomly selected for Sanger DNA sequencing analysis. Detailed sequences can be found in **Supplementary Figure S3**, and the inserted *amilGFP* sequence contained its own promoter. M, GeneRuler 1-kb DNA ladder (ThermoFisher Scientific).

Here we optimized the previously reported CRISPR/Cas9 system (Jiang et al., 2015), and developed a novel genome editing strategy using both the CRISPR/Cas9 system and an antibiotic resistance cassette (ARC). Firstly, an ARC is introduced nearby the target sites and the transformants are selected on antibiotic-containing plates. Secondly, the ARC sequence is employed as a cleavage target by Cas9 and then replaced with any edited DNA sequences by HDR (**Figure 1**). Because of the existence of multiple suitable PAM and protospacer sequences within the ARC, the procedure does not require PAM sequences within the genome. Consequently, we can modify the genome at any position *via* this technique.

## Results and Discussion

### Optimization of the CRISPR/Cas9 System

**FIGURE 2:**
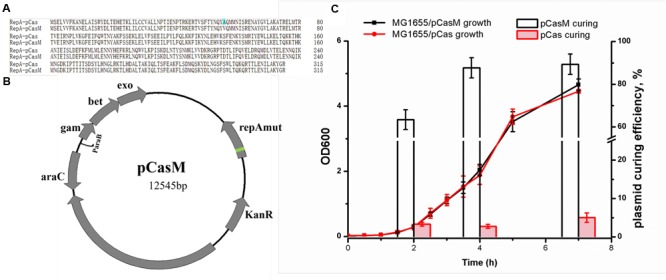
**Optimization of the CRISPR/Cas9 system. (A)** Sequence alignment between RepA101 expressed from pCas and pCasM. Alanine is mutated to valine at position 56. **(B)** Plasmid map for pCasM, which was similar to pCas (Jiang et al., 2015) but contained a RepA mutant (RepA_A56V_, refer to **A**). **(C)** Comparison of the plasmid curing rates between pCasM and pCas in *E. coli* MG1655 in the absence of antibiotics. To measure the growth rates, bacteria were grown in medium containing kanamycin.

At first, we employed the pCas system, which consists of both the *cas9* gene and a λ-Red recombineering system (Jiang et al., 2015). As pCas contains the temperature-sensitive replicon, repA101(Ts), the system needs to be operated at 30°C, which is time-consuming. We isolated a RepA mutant (RepA_A56V_), namely pCasM (**Figures 2A,B**), which both supported the culture of bacteria at 37°C and facilitated the rapid elimination of RepA_A56V_ plasmids in the absence of antibiotics (**Figure 2C**). This mutant was therefore employed for genome editing in this study and may also be used in other applications that require final elimination of the plasmids.

### Using CRISPR/Cas9 and Double-Stranded Donor DNA to Perform Precise Genome Editing

If the genomic sites to be edited are within the PAM or protospacer sequence, they can easily be manipulated because of the disruption of Cas9 recognition. However, if the sites to be edited are not within a protospacer or PAM sequence, conventional techniques cannot achieve clean editing because of PAM or protospacer modification. Hence, the two-step strategy employed here is superior in that it avoids the restriction of PAM availability. We took the editing of the *lacZ* gene in *E. coli* MG1655 as an example. At first, pCasM was transformed into MG1655 to allow for the induction of expression of the λ-Red recombineering system, and with its help, the ampicillin resistance gene (*bla*) was successfully inserted into *lacZ* by HDR. As a result of ampicillin selection, all transformants were positive colonies (**Supplementary Figure S2A**). Then, an sgRNA (sgRNA_bla) was designed to target the *bla* gene (scheme shown in **Supplementary Figure S1**) and the sgRNA plasmid was transformed into MG1655Δ*lacZ*::*bla* harboring pCasM. Jiang et al. (2015) acquired high editing efficiency by combining gRNA-expressing plasmid and donor DNA into a plasmid-borne editing template. Based on this, we attempted to co-transform the optimized two-plasmid CRISPR/Cas9 system along with linear donor dsDNA with the aim of achieving effective genome editing. When the donor dsDNA for HDR was absent, virtually no transformants were obtained (**Supplementary Figure S2B**), demonstrating that sgRNA_bla could be used to guide Cas9 to efficiently cleave the *bla* gene.

For the next step, co-transformation of sgRNA_bla and specific linear donor dsDNAs was conducted to accomplish genome editing, and in total three different donor dsDNAs were provided: 1) containing the ochre mutation in the *lacZ* initiation codon, 2) containing an inserted 6 × His encoding DNA sequence (CDS) after the ‘ATG’ initiation codon of *lacZ*, and 3) containing a fusion of the *amilGFP* gene to the 5’-end of the *lacZ* CDS (**Supplementary Figure S2B**). With the employment of the λ-Red recombineering system, HDR efficiency was largely increased. For each experiment, 20 colonies were randomly picked for colony PCR to confirm the genome editing efficiency, and high efficiency was found for all three editing experiments (**Figure 1B** and **Supplementary Figure S2B**). Sanger DNA sequencing further confirmed that all PCR-positive colonies contained the correct target DNA sequences (**Figure 1B** and **Supplementary Figure S3**).

Besides of sgRNA_bla, another two sgRNAs were designed for specifically targeting ARC (i.e., sgRNA_bla1 and sgRNA_bla2), both of which showed high editing efficiency (**Supplementary Figure S4**), demonstrating the robust of this strategy. In addition, different lengths of homologous arms of the donor dsDNAs were also tested. We noticed that a large number of colonies failed to be amplified with colony PCR with primers surrounding the target *lacZ* site, suggesting there were sequence rearrangements around the site, which was similar to the observation previously reported (Qi et al., 2013). Although the rearrangement ratio was found to be in a negative relationship with the length of the homologous arms, the positive ratio of the rest colonies that could be successfully PCR amplified was unaffected (**Supplementary Figure S5**).

**FIGURE 3:**
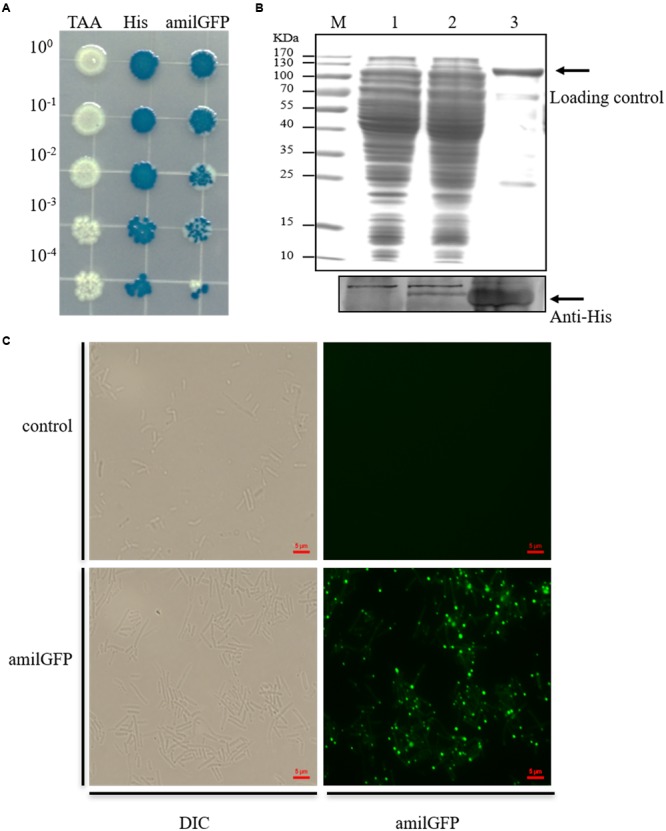
**Phenotypic analyses of the mutants. (A)** Serially diluted mutants were plated on LB containing IPTG and X-gal. In the TAA mutant, the initial codon (ATG) of *lacZ* was mutated to a stop codon (TAA), resulting in failure to express β-galactosidase. Consequently, the cells failed to degrade X-gal and remained white on the plate. His, N-terminal His-tagged mutant of *lacZ*. amilGFP, mutant with *amilGFP* fused to the 5’-end of the *lacZ* gene. **(B)** Western blot analysis to confirm the expression of the N-terminal His-tagged LacZ. About 30 μg of cell lysate and 1 μg of purified recombinant His-tagged LacZ were used for western blot analysis, and the same amounts of protein were used in separate SDS-PAGE, which was stained with Coomassie blue and used as a loading control. **(C)** Fluorescence microscopy detection of the expression of the AmilGFP-LacZ fusion protein.

Moreover, the engineered mutants were verified by phenotypic tests, which included blue/white colony screening, a western blot assay and fluorescence microscopy. As shown in **Figure 3**, the mutant containing the ochre mutation failed to turn blue when grown on LB plates supplemented with isopropyl β-D-1-thiogalactopyranoside (IPTG) and 5-bromo-4-chloro-3-indolyl-β-d-galactopyranoside (X-gal). For the mutant containing a His-tag on the N-terminal of LacZ, the western blot result clearly showed expression of the His-tagged LacZ. Similarly, expression of the AmilGFP-LacZ fusion protein could be observed by a fluorescence microscope in the *amilGFP-lacZ* mutant. Considering the expression of fluorescence by the mutant, we also employed FACS to precisely measure the recombination efficiency during the insertion of *amliGFP*, and the results showed that more than 93% edited cells successfully expressed AmilGFP (**Supplementary Figure S6**), which was consistent with the results of **Figure 1B**. In all mutants, the plasmid system was easily eliminated by culturing overnight with shaking in the absence of antibiotics (**Supplementary Figure S7**). In short, optimization of the CRISPR/Cas9 and two-step strategy, as described here, allowed for precise and efficient manipulation of the *E. coli* genome.

### Genome Editing of Essential Genes

**FIGURE 4:**
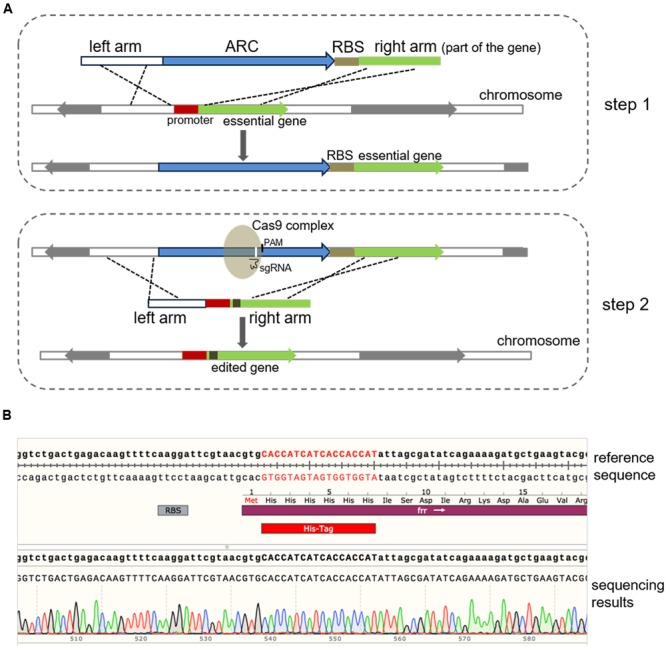
**Modification of essential genes using a modified two-step strategy. (A)** Schematic chart illustrating the modified two-step strategy for editing an essential gene with or without an independent promoter. The whole procedure was similar to the method described above (**Figure 1**) except that a ribosome binding site sequence (BBa_J61101 in this study) was linked to the ARC. To edit the 5′-end or the internal sequence of an essential gene, the ARC-ribosome binding site should be inserted in front of the CDS to ensure efficient expression of the gene. **(B)** Modification of the *frr* gene (*b0172*), an essential gene in *E. coli*. The 6 × His-tag-encoding DNA sequence was successfully inserted after the initial codon of *frr*, which was verified by Sanger DNA sequencing. Sequence alignment was performed using the SnapGene software (GSL Biotech).

To edit the 3′-end of an essential gene, similar procedures can be employed as mentioned above, where an ARC can be inserted nearby the stop codon but outside of the CDS. Whereas, to edit the 5′-end or the internal region of an essential gene, with or without an independent promoter, a modified version of this method can be used, where a synthetic ribosome binding site (BBa_J61101) is placed after the ARC, which thus allows for the normal transcription and translation of the essential gene (**Figure 4A**). To test this system, the *frr* gene, which is an essential gene encoding a ribosome releasing factor (Janosi et al., 1994), was selected, and according to the Sanger sequencing results, the His-tag DNA sequence was successfully inserted after the translation initiation codon in the *frr* gene (**Figure 4B**). To the best of our knowledge, this is the first report describing the genome editing of essential genes using CRISPR/Cas9 in *E. coli*.

### The Advantages of the Newly Developed Method

It is worth mentioning that the ARC is employed in our method as both a positive selection marker during the first step and a negative selection marker (the cleavage target of Cas9) during the second step. Although the Cas9-assisted dsDNA break may increase the editing efficiency of the first step, it is not necessary when the λ-Red recombineering system is employed. Therefore, as shown in **Supplementary Figure S8**, our method provides a simplified experimental procedure and is time saving.

**FIGURE 5:**
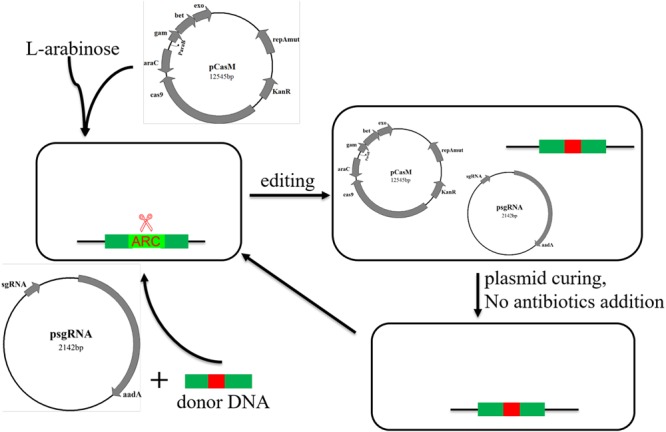
**An overview of successive genome editing with the new psgRNA and pCasM two-plasmid system**.

Recently, the Cas9-mediated two-step methods have been reported that allow for genome editing (including point mutations). Specifically, during the first step, artificial protospacer sequences are introduced to replace the original protospacer near the target sites using the CRISPR/Cas9 system. Then, through employing a second sgRNA that recognizes the introduced artificial protospacer sequence, the target sites will be mutated, and meanwhile the artificial PAM or protospacer sequence will be back-mutated to its original sequence, generating a clean mutant with only the target sites mutated (Biot-Pelletier and Martin, 2016). Wang et al. (2016) reported a similar approach. The PAM or protospacer used in the first step was modified or deleted, whereas the artificial PAM was created at the target site. Then in a second step, through CRISPR/Cas9 editing, the modified or deleted PAM or protospacer sequence was changed back into its original sequence and the artificial PAM was changed into the desired mutation (Wang et al., 2016). Although these methods are effective, their first steps require a proper PAM site near to the target sites, which may limit their application. That is, these methods cannot edit target sequences without a PAM sequence in the vicinity. Moreover, to avoid continuous cutting of the modified genome, the sgRNA-expressing plasmid must be cured before the second step of genome manipulation, which could be time consuming. By contrast, our strategy (**Figure 5**) allows for convenient and prompt curing of the sgRNA-expressing plasmid, saving much time.

In summary, we describe an efficient two-step genome editing approach that integrates both the CRISPR system and an ARC. A specific modification of the target genome site can be generated with ease by supplying a template DNA containing the desired mutation. As the method is versatile and does not require any PAM sites in the genome, it can therefore be used to conveniently edit any DNA sequences.

## Materials and Methods

### Strains, Plasmids, and Growth Conditions

*Escherichia coli* strain DH10B was used for plasmid cloning and strain MG1655 was used for genome editing analysis. Plasmids pTarget and pCas were kindly provided by Prof. Sheng Yang (Jiang et al., 2015). Unless indicated, all *E. coli* strains were grown in Luria–Bertani (LB) medium and incubated at 37 °C. All strains and plasmids used in this study are listed in **Table 1** and all primers are listed in **Table 2**.

**Table 1:** Strains and plasmids used in this study.

Strain or Plasmid	Relevant properties	Sources
**Strain**		
*E. coli* DH10B	F^-^ *endA1 deoR*^+^ *recA1 galE15 galK16 nupG rpsL* Δ*(lac)X74* φ80*lacZΔM15 araD139* Δ*(ara,leu)7697 mcrA* Δ*(mrr-hsdRMS-mcrBC)* Str^R^ λ^-^	Invitrogen
*E. coli* MG1655	F- lambda- *ilvG*- *rfb*-50 *rph*-1	Lab stock
MG1655Δ*lacZ*::*bla*	MG1655 with *lacZ* inserted with *bla* gene, Amp^r^	This study
MG1655-*lacZ*(TAA)	MG1655 with the initial codon of *lacZ* mutated to ‘TAA,’ plasmids cured	This study
MG1655-*lacZ*(His)	MG1655 with the His-tag DNA sequence inserted after the initial codon of *lacZ*, plasmids cured	This study
MG1655-*lacZ*(*amilGFP*)	MG1655 with the *amilGFP* CDS fused to the 5′-end of *lacZ*, plasmids cured	This study
MG1655Δ*frr*::*bla*	MG1655 with the *bla* gene and RBS inserted to the front of *frr* gene, Amp^r^	This study
MG1655-*frr*(His)	MG1655 with the His-tag DNA sequence inserted after the initial codon of *frr*, plasmids cured	This study
**Plasmid**		
pUC18	pUC, *bla*	Lab stock
pSY1071	pSY1069 carrying amilGFP (BBa_K592010)	Li et al., 2016
pTarget	pMB1, *aadA*, sgRNA-fts	Jiang et al., 2015
pCas	repA101(Ts), kan^r^, P_cas_-cas9, P_araB_-Red, lacI^q^, P_trc_-sgRNA-pMB1; temperature-sensitive replication vector	Jiang et al., 2015
pCasM	A derivative plasmid from pCas, harboring a mutant repA101 (RepA_A56V_)	This study
psgRNA_bla	A derivative plasmid from pTarget, expressing an sgRNA targeting *bla* gene	This study

**Table 2:** Oligoes used in this study.

Oligo name	Sequences (5′-3′)	Description
**Mutation of *lacZ***		
LacZ-UF LacZ-UR Amp-lacZF Amp-lacZR LacZ-DF LacZ-DR	agcatctggtcgcattgggtca acgacgttgtaaaacgacggccagt ctggccgtcgttttacaacgtcgtgacgaaagggcctcgtgatacg ttaccaatgcttaatcagtgaggca ctcactgattaagcattggtaagactgggaaaaccctggcgttac cttccagataactgccgtca	LacZ-UF/LacZ-UR: amplification of the upstream homologous arm; Amp-lacZF/Amp-lacZR: amplification of the *bla* gene from pUC18; LacZ-DF/ LacZ-DR: amplification of the downstream homologous arm; The above three fragments were assembled *via* fusion PCR to obtain a fragment for insertion of the ARC to the *lacZ* gene in MG1655.
LacZ-UF lacZ-mutation-UR lacZ-mutation-DF LacZ-DR	The same sequences as shown above gtaatcatggtttaagctgtttcct Atttcacacaggaaacagcttaaaccatga The same sequences as shown above	LacZ-UF/lacZ-mutation-UR: amplification of the upstream homologous arm; lacZ-mutation-DF/LacZ-DR: amplification of the downstream homologous arm; The above two fragments were assembled *via* fusion PCR to obtain a fragment for mutation of the initial codon of *lacZ* gene to ‘TAA.’
LacZ-UF5 LacZ-DR5 LacZ-UF3 LacZ-DR3 LacZ-UF1 LacZ-DR1	ggcatcgttcccactgcgatgc cgactgtcctggccgtaaccga ccgcttgctgcaactctctcag gatgggcgcatcgtaaccgtgc tagctcactcattaggcacccc aagggggatgtgctgcaaggcg	LacZ-UF5/LacZ-DR5: amplification of 500-bp homologous arms, and the amplicons were then used as the templates for in-fusion PCR to obtain the donor DNA for recombination to change the *lacZ* start codon to ‘TAA’; Similarly, LacZ-UF3/LacZ-DR3 and LacZ-UF1/LacZ-DR1 were used for amplification of 300-bp and 100-bp homologous arms, respectively.
LacZ-UF lacZ-insHis-UR lacZ-insHis-DF LacZ-DR	The same sequences as shown above gtgaatccgtaatcatggtgtggtggtggtggtggtgcatagctgtttcctgtgtg cacacaggaaacagctatgcaccaccaccaccaccacaccatgattacggattcac The same sequences as shown above	LacZ-UF/lacZ-insHis-UR: amplification of the upstream homologous arm; lacZ-insHis-DF/LacZ-DR: amplification of the downstream homologous arm; The above two fragments were assembled *via* fusion PCR to obtain a fragment for insertion of the ‘His-tag’ sequence after the initial codon of *lacZ* gene.
LacZ-UF lacZ-insGFP-UR amilGFP-F amilGFP-R lacZ-insGFP-DF LacZ-DR	The same sequences as shown above agctgtttcctgtgtgaaattgtt taacaatttcacacaggaaacagctcatcggtaagttattcttgaca tttaaccttcaaagggttaacat tgttaaccctttgaaggttaaaatgaccatgattacggattcact The same sequences as shown above	LacZ-UF/lacZ-insGFP-UR: amplification of the upstream homologous arm; amilGFP-F/ amilGFP-R: amplification of the amilGFP gene from plasmid pSY1071; lacZ-insGFP-DF/LacZ-DR: amplification of the downstream homologous arm; The above three fragments were assembled *via* fusion PCR to obtain a fragment for insertion of the *amilGFP* gene to the 5′-end of the *lacZ* gene.
AmpsgRNA-F AmpsgRNA-R	TCCTAGGTATAATACTAGTaaagatgctgaagatcagtt GTTTTAGAGCTAGAAATAGC Actagtattatacctaggactgagctagctgtcaag	Construction of psgRNA_bla
AmpsgRNA-F1 AmpsgRNA-R	TCCTAGGTATAATACTAGTacactattctcagaatgact GTTTTAGAGCTAGAAATAGC The same sequences as shown above	Construction of psgRNA_bla1
AmpsgRNA-F2 AmpsgRNA-R	TCCTAGGTATAATACTAGTaatagacagatcgctgagat GTTTTAGAGCTAGAAATAGC The same sequences as shown above	Construction of psgRNA_bla2
lacZ_Amp-seq	ttaccaatgcttaatcagtgaggca	The sequencing primer to confirm that ARC has been inserted to the *lacZ* gene.
**Modification of *frr***		
frr-UF frr-UR Amp-frrF Amp-frrR frr-DF frr-DR	agcttgcctgcgtggtatcg cctcacgtacttttgtacgc gcgtacaaaagtacgtgaggGACGAAAGGGCCTCGTGATAC catctagtgggtcctgtctttttaccaatgcttaatcagtgaggcacctatc AAAGACAGGACCCACTAGATGgtgattagcgatatcagaaaagatgctg actttgtcgttcgcgtcacg	frr-UF/frr-UR: amplification of the upstream homologous arm; Amp-frrF/Amp-frrR: amplification of the *bla* gene from pUC18; frr-DF/frr-DR: amplification of the downstream homologous arm; The above three fragments were assembled *via* fusion PCR to obtain a fragment for insertion of the ARC to the *frr* gene in MG1655.
frr_Amp-seq	tggtcctgcaactttatccg	The sequencing primer to confirm that ARC has been inserted to the *frr* gene.
frr-UF frr-insHis-UR frr-insHis-DF frr-DR	The same sequences as shown above cacgttacgaatccttgaaaacttg gttttcaaggattcgtaacgtgCACCATCATCACCACCAT attagcgatatcagaaaagatgctg The same sequences as shown above	frr-UF/frr-insHis-UR: amplification of the upstream homologous arm; frr-insHis-DF/frr-DR: amplification of the downstream homologous arm; The above two fragments were assembled *via* fusion PCR to obtain a fragment for insertion of the ‘His-tag’ sequence after the initial codon of *frr* gene.

*lacZ-UF/lacZ-DR were used for sequencing the mutation of lacZ gene, including ‘TAA’ mutation, His-Tag insertion and *amilGFP* insertion. For *amilGFP* insertion, primer amilGFP-F was also used for sequencing analysis. For frr modification, frr-UF/frr-DR were used for sequencing analysis of the insertion of His-Tag in *frr* gene.*

### Optimization of the CRISPR/Cas9 System

First, we adopted the primary pCas system, as pCas requires a temperature of 30°C. However, to optimize the efficiency of the system, we attempted to culture bacteria at 37°C with several inoculations and a RepA mutant was obtained, which was designated pCasM.

### Procedures for the Insertion of an ARC Nearby the Target Site

The optimized plasmid pCasM was transformed into *E. coli* MG1655 and a transformant was used to prepare electrocompetent cells. The expression of the λ-Red recombinases was induced by addition of 10 mM arabinose. When the OD_600_ reached 0.6, cells were pre-chilled on ice for about 10 min before being washed three times with 10% cold glycerol. About 5 ml of cells were finally concentrated to 50 μl for each electroporation reaction.

Both upstream and downstream homologous arms were PCR amplified with primers listed in **Table 2**, and were then fused with the ampicillin resistance cassette (the ARC used in this study) by fusion PCR. About 50 μl of electrocompetent cells were mixed with 300 ng of the ARC, and electroporation was performed in a 2-mm cuvette using the GenePulser Xcell^TM^ (Bio-Rad, USA; 2.5 kV, 200 Ω). Transformants were suspended immediately in 1 ml of ice-cooled LB liquid, and recovered by shaking at 37°C for 1 h before being spread onto LB agar medium containing ampicillin (100 μg/ml). After being incubated overnight at 37°C, transformants were verified by colony PCR and DNA sequencing, using the primers listed in **Table 2**.

### Genome Editing

To construct psgRNA_bla, a pair of primers were used for PCR amplification using pTarget (Jiang et al., 2015) as the template. A 20-bp spacer sequence (N20) was specifically designed to target the ARC, and this was incorporated into the primers. The amplicon was digested with *Dpn*I (NEB, USA) to remove the template, and was then transformed into DH10B to obtain the desired psgRNA plasmid, namely psgRNA_bla, followed by verification by Sanger DNA sequencing.

Donor dsDNA fragments usually contained a 300–500 bp homologous arm on each side. For mutation of the *lacZ* initiation codon and labeling with an N-terminal His-tag, DNA sequences of “TAA” and “CACCACCACCACCAC” were directly incorporated into primers to amplify the upstream and downstream homologous arms. For the fusion of *amilGFP* to *lacZ*, two homologous arms and the *amilGFP* CDS sequence were separately amplified and then assembled together by fusion PCR. For labeling of the *frr* gene with the N-terminal His-tag, the DNA sequence of “CACCATCATCACCACCAT” was directly incorporated into the primer for amplification of the downstream homologous arm. Both upstream and downstream homologous arms were then assembled by fusion PCR. All PCR products were purified by gel electrophoresis prior to electroporation.

Cells harboring the ARC and pCasM were used for the preparation of electrocompetent cells, following the same procedure as described above. The electroporation parameters were the same as described, and 200 ng psgRNA plasmid and 300 ng donor dsDNA were added to each electroporation reaction. Cells were recovered at 37°C for 1 h before being plated on LB agar containing kanamycin (50 μg/ml) and spectinomycin (300 μg/ml) and incubated overnight at 37°C. Transformants were verified by colony PCR and subsequent Sanger DNA sequencing.

### Phenotypic Analyses

#### Blue/White Screening Assay

Cells were grown overnight at 37°C in LB liquid medium, and were then diluted to OD_600_ = 0.1. Ten-fold serial dilution was performed, and 2.5 μl of cells of different concentrations were plated on LB agar plates containing 0.1 mM IPTG and 40 μg/ml X-gal. After being incubated at 37°C overnight, imaging of the plates was performed.

#### Western Blot Assay

To examine the expression of His-tagged LacZ, 50 ml of cells were induced for the expression of His-LacZ by the addition of 0.1 mM IPTG when the OD_600_ reached 0.6. Cells were harvested, resuspended in lysis buffer (20 mM Tris–HCl pH 7.6, 0.1 M NaCl) and lysed by sonication, and supernatants were obtained by centrifugation. To purify His-LacZ, 1 ml of Ni-NTA resin (GE Healthcare, USA) was used, and purification was performed following the manufacturer’s instructions.

Protein samples were separated by 12% SDS–PAGE and visualized by Coomassie blue staining. For the western blot assay, the gels were transferred to nitrocellulose membrane that was blocked with 5% dry skim milk in TBST (0.05% Tween-20 in TBS buffer) and incubated with the primary mouse polyclonal antibody against His-tags (1:5000; Abmart, China). HRP-conjugated goat anti-mouse IgG antibody (1:5000; Abmart) was used as the secondary antibody. Enhanced chemiluminescence blotting reagent (GE Healthcare) was used for detection and imaging was performed using a ImageQuant LAS 4000 mini (GE Healthcare).

#### Fluorescence Microscopy

To confirm the expression of the AmilGFP-LacZ fusion protein, the *amilGFP-lacZ* mutant was induced as described above. Then, fresh cells (OD_600_ = 1.0) were diluted 100-fold and 2.5 μl of diluted cells were dropped onto a slide and covered with a coverslip. Images were taken using a fluorescent microscope (Nikon, Japan) with differential interference contrast equipped with optical filter sets, and with excitation at 490 nM and emission at 520 nM for green fluorescence.

#### Flow Cytometry Analysis

All flow cytometry (FCM) analyses were performed using a MoFlo XDP Flow-Cytometer (Beckman Coulter, USA) equipped with an argon laser at 488 nm. Forward and side scatters were gated on the major population of cells with normal sizes. For each sample, at least 1,000,000 cells were analyzed, and the relative proportion of cells with different fluorescent profiles were quantified using the Summit Software (version 5.2) with results expressed as the mean fluorescence intensity.

### Plasmid Curing

To eliminate psgRNA and pCasM plasmid, a positive colony was selected and inoculated into 5 ml liquid LB medium with no antibiotics for overnight culture. Then, cells were streaked onto LB agar to allow for the formation of single colonies, which were then checked by a growth test on plates containing kanamycin (50 μg/ml), spectinomycin (200 μg/ml) or no antibiotics. When a colony was sensitive to both antibiotics, it would have lost both plasmids and could therefore be used for further analysis. For curing of the psgRNA plasmid, an alternative method was to grow the cells harboring psgRNA in liquid LB medium with IPTG (0.2 mM) to induce the expression of an sgRNA specifically targeting the psgRNA plasmid, which would then lead to the cleavage of psgRNA by Cas9 expressing from pCasM plasmid.

## Author Contributions

JW designed the experiments. HZ and Q-XC performed all the experiments. All the authors wrote and revised the manuscript.

## Conflict of Interest Statement

The authors declare that the research was conducted in the absence of any commercial or financial relationships that could be construed as a potential conflict of interest.

## Supplementary Material

FIGURE S1 **Sketch of sgRNA and donor DNA cassette.** The *lacZ* gene was used as an example in this study. ARC (*bla* here) was inserted into *lacZ*, and sgRNA coupled with Cas9 targeted *bla* to generate a site-specific dsRNA break. When the donor dsDNA for homologous recombination was provided, genome manipulation was completed. DSB, Site-specific dsRNA break; HDR, homologous recombination. Dark green DNA: any mutation; purple DNA: upper and lower arms.

FIGURE S2 **Genome editing efficiency of the two-step procedure. (A)** The efficiency of the first step of the genome editing experiments, where an ARC (*bla* gene) was inserted into the *lacZ* gene. More than 2,000 colonies were obtained, and all transformants showed successful integration of the *bla* gene (100% positivity) with the assistance of ampicillin selection. MG1655 harboring pCasM without transformation of the *bla* recombination fragment was used as a negative control. **(B)** The efficiency of the second step, where the *bla* gene was replaced with the edited *lacZ* gene, including a TAA mutation, N-terminal His-tag insertion and N-terminal *amilGFP* insertion. Homologous arms of 700 bps in length were employed for HR in both the first and the second steps. MG1655Δ*lacZ*::*bla* harboring pCasM was transformed with psgRNA_bla but without the recombination fragment was employed as a negative control.

FIGURE S3 **Alignment analysis of the wild-type and edited *lacZ* sequences, including the TAA mutation (A)**, the His-tag insertion **(B)**, and the *amilGFP* sequence insertion **(C)**. The inserted *amilGFP* sequence shown by a blue background contained its own promoter. Primers used for amplification of both wild-type and edited sequences, as well as the primers used for sequencing, are shown in **Table 2**. Different sequences are indicated by a blue background.

FIGURE S4 **Genome editing efficiency using different psgRNA_bla plasmids. (A)** Schematic chart representing the tested three sgRNAs targeting to different positions of ARC. **(B)** Verification of the edited mutants by colony PCR. Correct sizes were indicated by arrows and the negative control was indicated by ‘C.’

FIGURE S5 **Genome editing efficiency using donor DNA with different lengths of homologous arms.** Three different lengths of 500, 300, and 100 bps were employed and three independent transformation was performed. Totally, 20 colonies of each test were randomly selected for colony PCR to verify the recombination efficiency. For shorter homologous arms, a large number of colonies failed to be amplified, which might be caused by sequence rearrangements. The recombination efficiency was calculated as the ratio of positive colonies/successfully amplified colonies.

FIGURE S6 **Flow cytometry (FCM) analyses to check the expression of AmilGFP fusion protein. (A)** MG1655Δ*lacZ*::*bla* was used as the negative control. **(B)** MG1655-*lacZ*(*amilGFP*) was used as the positive control. **(C)** Measurement of the recombination efficiency through detection of the AmilGFP expression. After electroporation of the donor DNA containing the AmilGFP gene and homologous fragments, cells were recovered and then inoculated into 5 ml fresh LB medium with antibiotics added to allow for overnight culture in 37°C shaker, followed by FCM analyses.

FIGURE S7 **Self-curing of plasmids psgRNA and pCasM in *E. coli* including the TAA mutation (A)**, the His-tag insertion **(B)**, and the *amilGFP* sequence insertion **(C)**. After overnight culture by shaking in liquid LB medium without antibiotics, cells were diluted and plated on LB agar without antibiotics. Single colonies were then plated on LB agar containing different antibiotics; LB agar containing no antibiotics was included as a control. Cells that lost psgRNA were spectinomycin sensitive, and cells that lost pCasM were kanamycin sensitive.

FIGURE S8 **Step-by-step schematic diagram of iterative genome editing.** The temperature required for each step was indicated in red.
