# Supplementary figures and images for: A Novel and Efficient Method for Bacteria Genome Editing Employing both CRISPR/Cas9 and an Antibiotic Resistance Cassette
Source: Front Microbiol. 2017 May 5;8:812. doi: 10.3389/fmicb.2017.00812 (PMC5418352; doi:10.3389/fmicb.2017.00812)

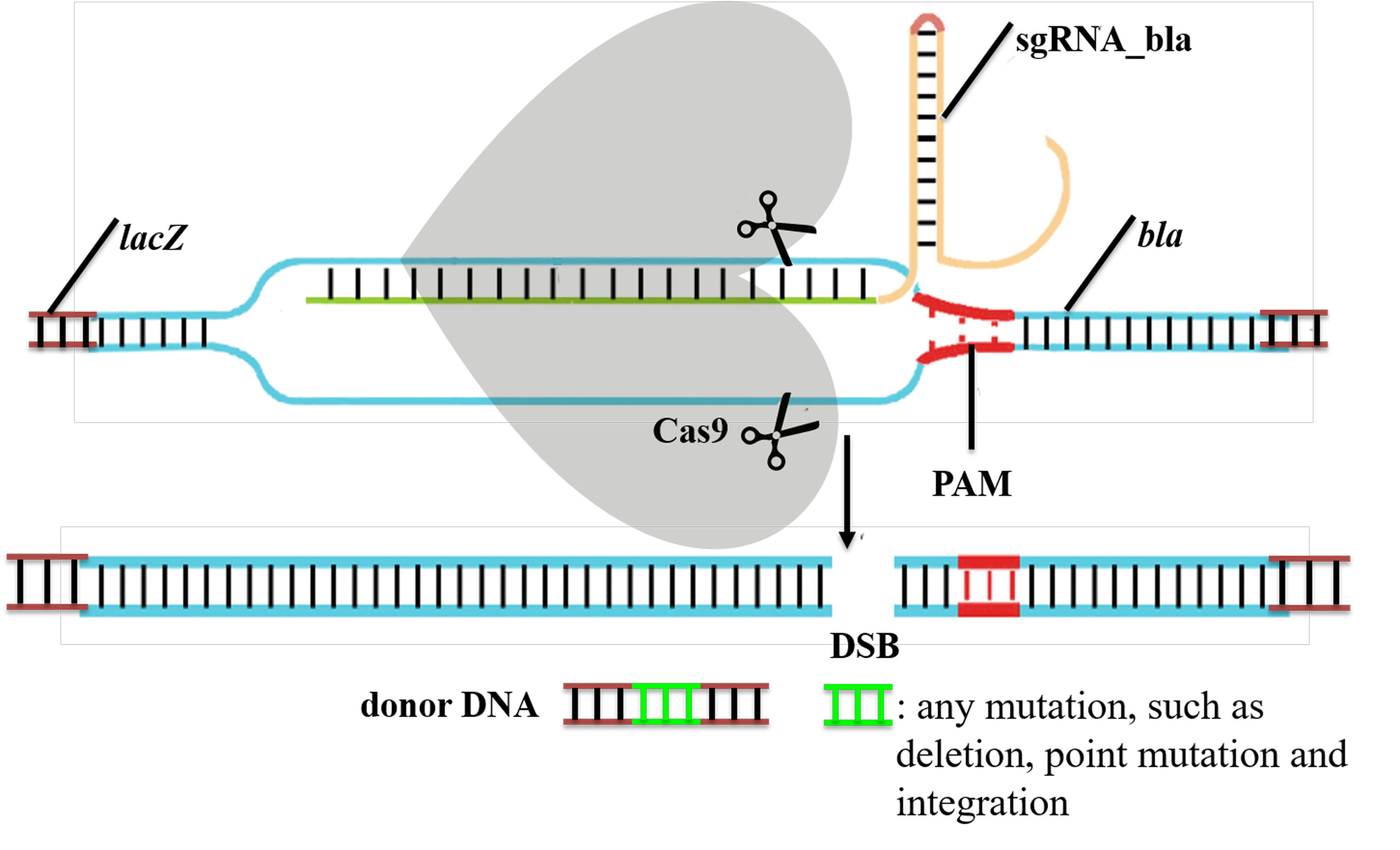

Supplement: FIGURE S1 — Sketch of sgRNA and donor DNA cassette. The lacZ gene was used as an example in this study. ARC (bla here) was inserted into lacZ, and sgRNA coupled with Cas9 targeted bla to generate a site-specific dsRNA break. When the donor dsDNA for homologous recombination was provided, genome manipulation was completed. DSB, Site-specific dsRNA break; HDR, homologous recombination. Dark green DNA: any mutation; purple DNA: upper and lower arms. [file Image_1.TIF]

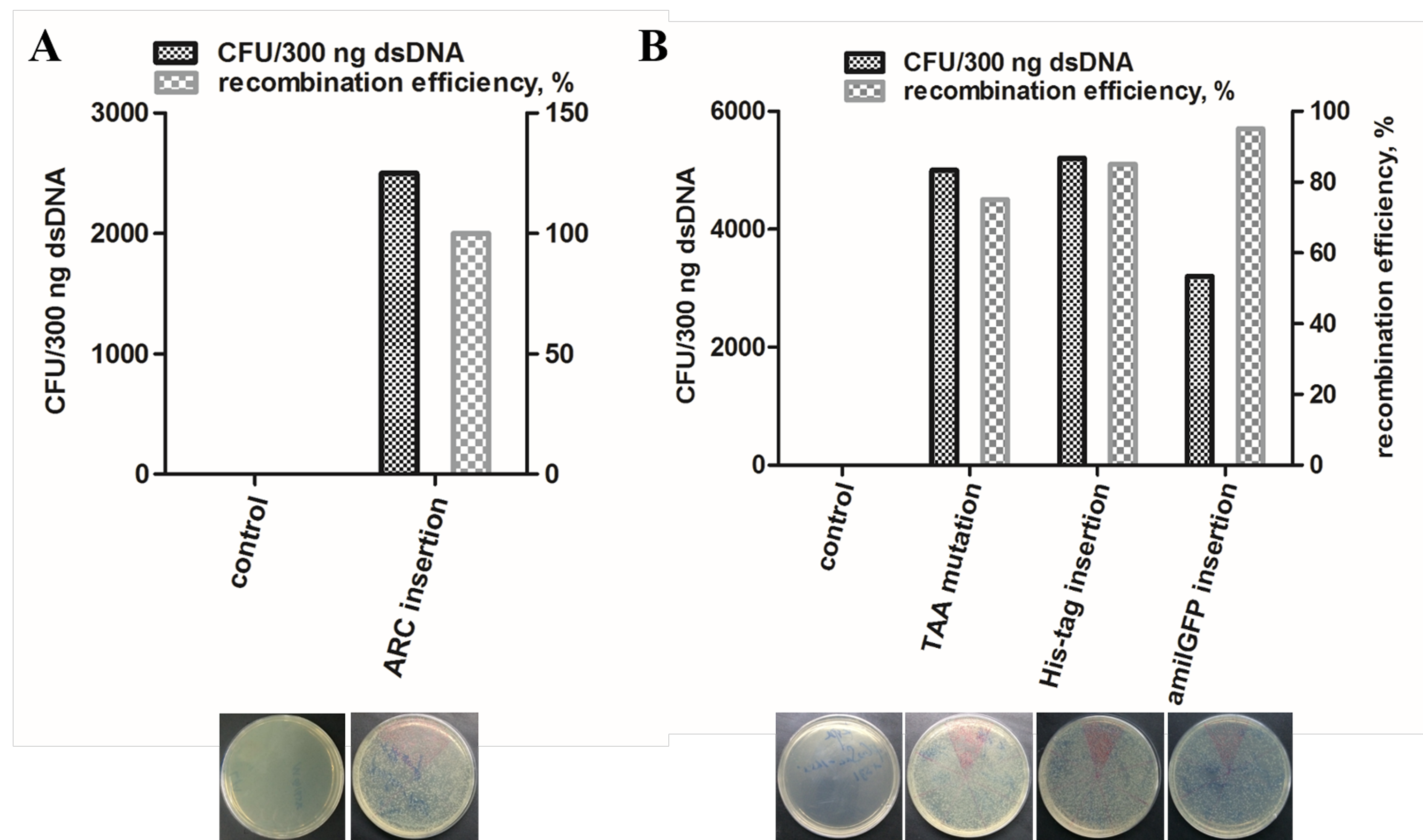

Supplement: FIGURE S2 — Genome editing efficiency of the two-step procedure. (A) The efficiency of the first step of the genome editing experiments, where an ARC (bla gene) was inserted into the lacZ gene. More than 2,000 colonies were obtained, and all transformants showed successful integration of the bla gene (100% positivity) with the assistance of ampicillin selection. MG1655 harboring pCasM without transformation of the bla recombination fragment was used as a negative control. (B) The efficiency of the second step, where the bla gene was replaced with the edited lacZ gene, including a TAA mutation, N-terminal His-tag insertion and N-terminal amilGFP insertion. Homologous arms of 700 bps in length were employed for HR in both the first and the second steps. MG1655ΔlacZ::bla harboring pCasM was transformed with psgRNA_bla but without the recombination fragment was employed as a negative control. [file Image_2.TIF]

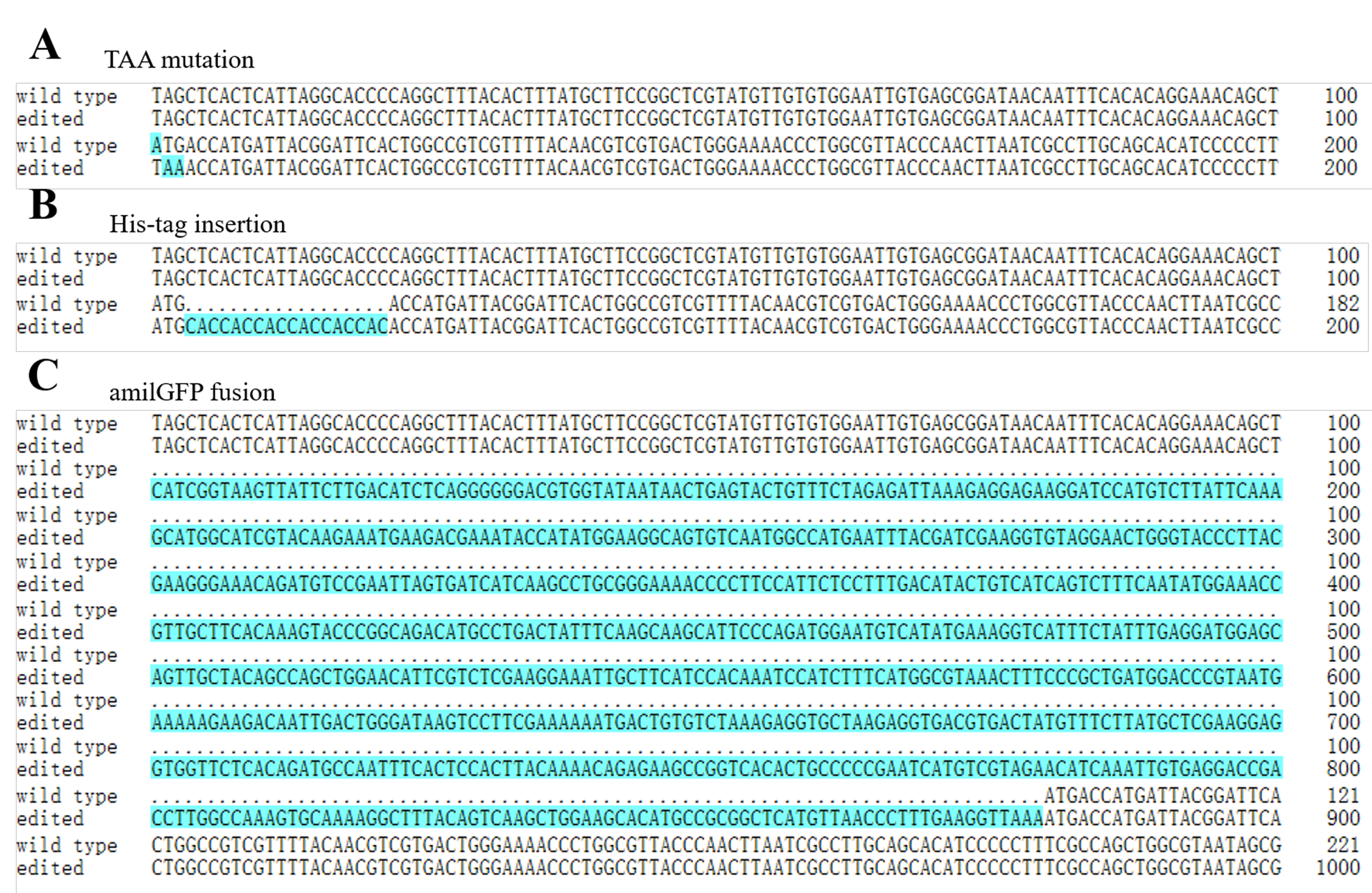

Supplement: FIGURE S3 — Alignment analysis of the wild-type and edited lacZ sequences, including the TAA mutation (A), the His-tag insertion (B), and the amilGFP sequence insertion (C). The inserted amilGFP sequence shown by a blue background contained its own promoter. Primers used for amplification of both wild-type and edited sequences, as well as the primers used for sequencing, are shown in Table 2. Different sequences are indicated by a blue background. [file Image_3.TIF]

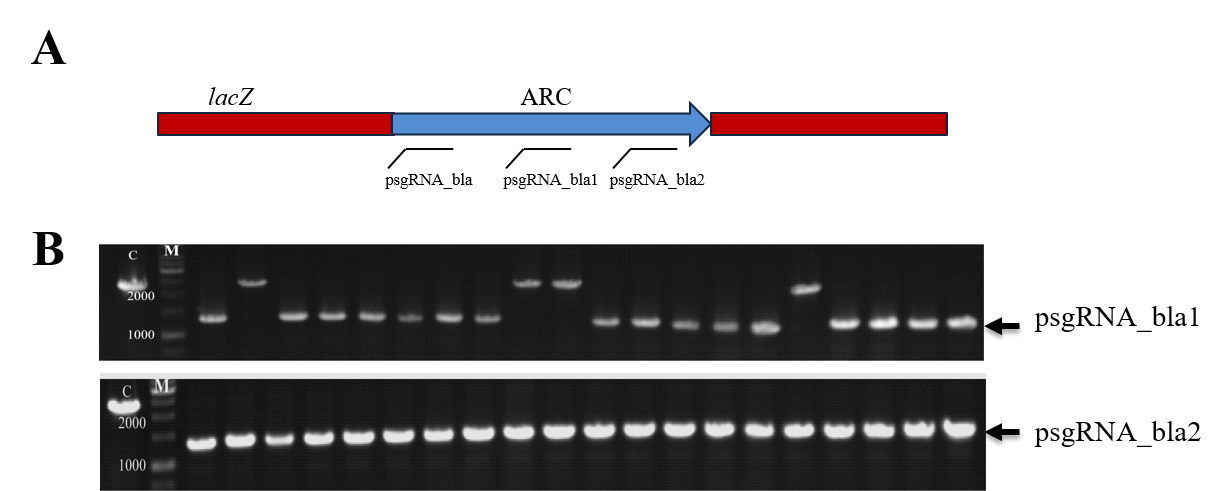

Supplement: FIGURE S4 — Genome editing efficiency using different psgRNA_bla plasmids. (A) Schematic chart representing the tested three sgRNAs targeting to different positions of ARC. (B) Verification of the edited mutants by colony PCR. Correct sizes were indicated by arrows and the negative control was indicated by ‘C.’ [file Image_4.TIF]

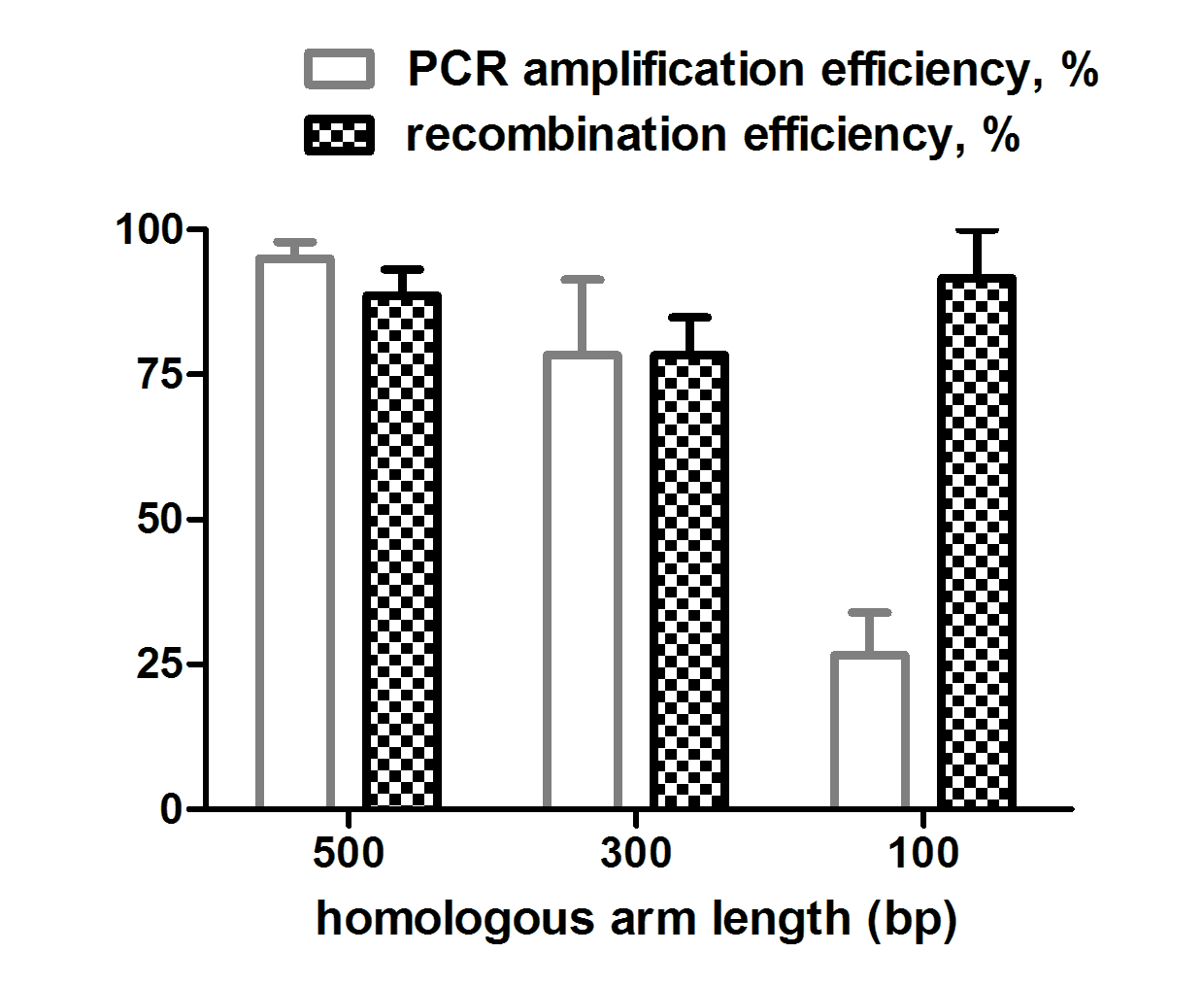

Supplement: FIGURE S5 — Genome editing efficiency using donor DNA with different lengths of homologous arms. Three different lengths of 500, 300, and 100 bps were employed and three independent transformation was performed. Totally, 20 colonies of each test were randomly selected for colony PCR to verify the recombination efficiency. For shorter homologous arms, a large number of colonies failed to be amplified, which might be caused by sequence rearrangements. The recombination efficiency was calculated as the ratio of positive colonies/successfully amplified colonies. [file Image_5.TIF]

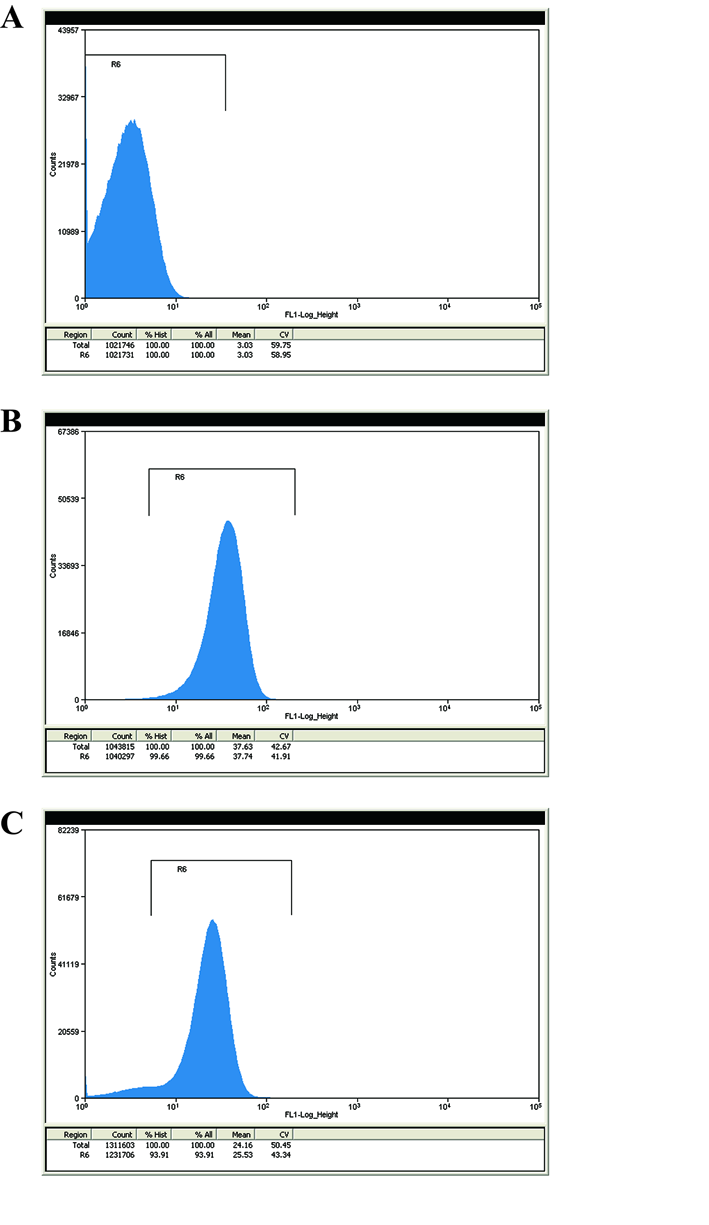

Supplement: FIGURE S6 — Flow cytometry (FCM) analyses to check the expression of AmilGFP fusion protein. (A) MG1655ΔlacZ::bla was used as the negative control. (B) MG1655-lacZ(amilGFP) was used as the positive control. (C) Measurement of the recombination efficiency through detection of the AmilGFP expression. After electroporation of the donor DNA containing the AmilGFP gene and homologous fragments, cells were recovered and then inoculated into 5 ml fresh LB medium with antibiotics added to allow for overnight culture in 37°C shaker, followed by FCM analyses. [file Image_6.TIF]

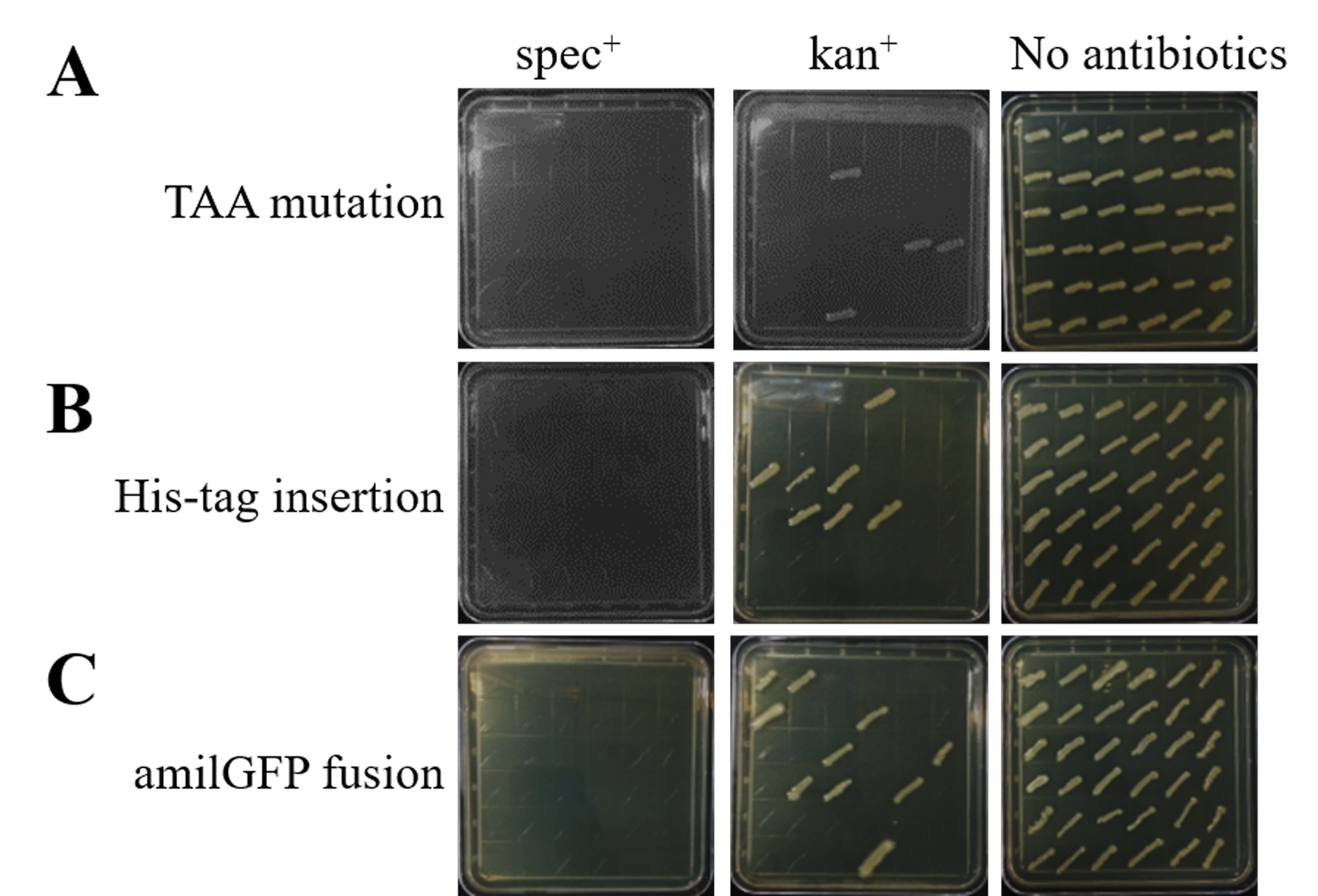

Supplement: FIGURE S7 — Self-curing of plasmids psgRNA and pCasM in E. coli including the TAA mutation (A), the His-tag insertion (B), and the amilGFP sequence insertion (C). After overnight culture by shaking in liquid LB medium without antibiotics, cells were diluted and plated on LB agar without antibiotics. Single colonies were then plated on LB agar containing different antibiotics; LB agar containing no antibiotics was included as a control. Cells that lost psgRNA were spectinomycin sensitive, and cells that lost pCasM were kanamycin sensitive. [file Image_7.TIF]

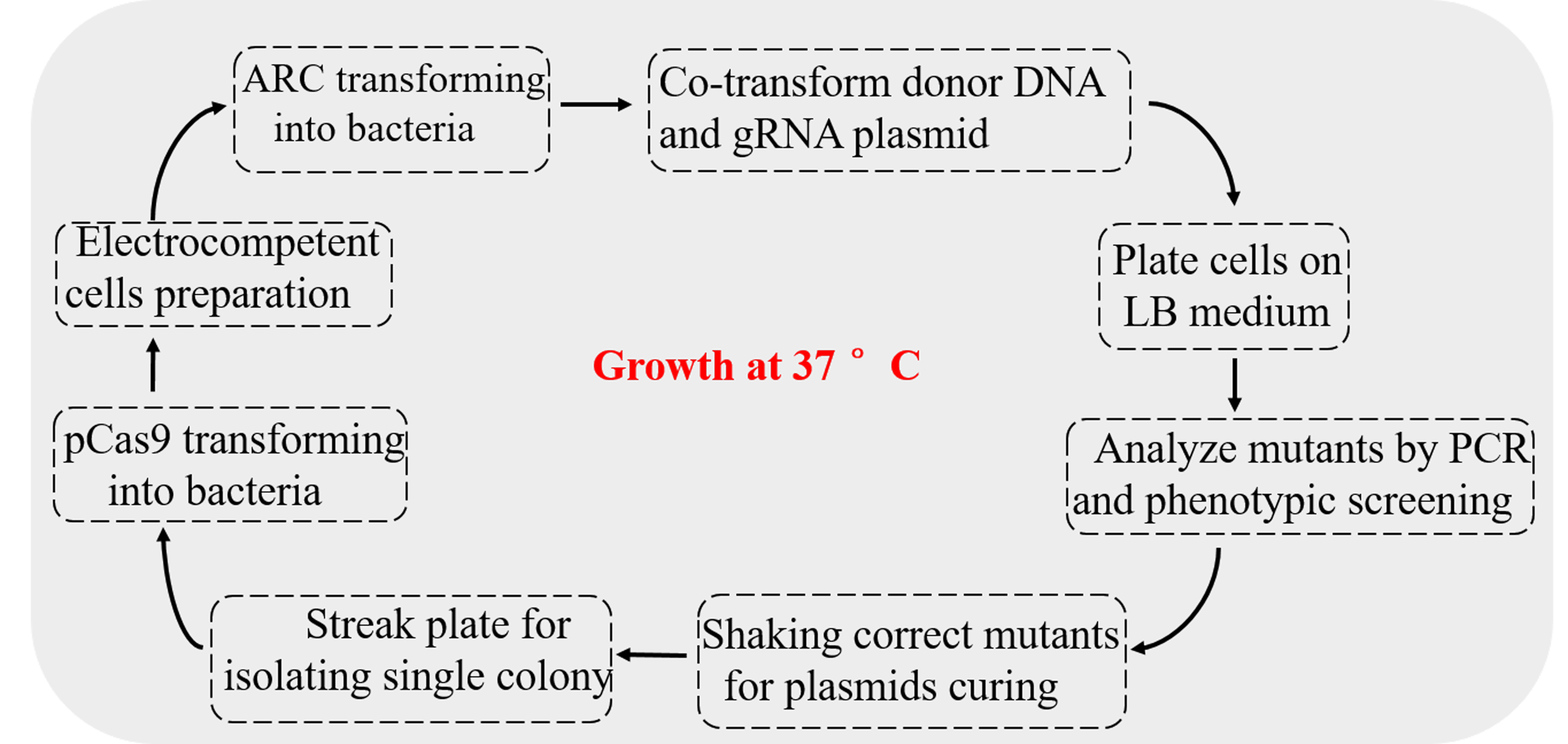

Supplement: FIGURE S8 — Step-by-step schematic diagram of iterative genome editing. The temperature required for each step was indicated in red. [file Image_8.TIF]
